# Buffalo Academy of Medicine

**Published:** 1905-07

**Authors:** Franklin W. Barrows


### SOCIETY PROCEEDINGS.

### Buffalo Academy of Medicine

*Section on Medicine, April 11, 1905.*

REPORTED BY FRANKLIN W. BARROWS, M. D., Secretary.

THE regular meeting of the medical section was held Tuesday evening, April 11, 1905, at the academy rooms in the Public Library building. The meeting was called to order at 9 o'clock by the chairman, Dr. Allen A. Jones. The minutes of the last meeting were read and approved.

DR. HENRY DWIGHT CHAPIN, professor of the diseases of children, in the Post-Graduate Medical School, New York, presented a paper entitled,

### THE FUTURE VIEWPOINT AND PRACTICE OF INFANT FEEDING.

*Author's Abstract.*

The attempts at placing infant feeding on a scientific basis during the past twenty years have been devoted principally to altering cow's milk so that it should resemble human milk in its chemical properties. Peptonising milk, diluting it and adding cream, sugar and some alkali, sterilisation and pasteurisation have been advocated, but the problem of making human milk has not yet been solved. It is now recognised that studies must be made in other directions. The mother supplies the fetus and infant with food in six different forms from conception to weaning, and milk is one of these forms. The object of changing the form of the food, is to make it suitable for the infant as it develops. The infant at birth is not completely formed, and while supplied with milk its digestive apparatus undergoes a transformation that fits it for solid food. Mother's milk is an elastic food, in that when it comes in contact with the infant's gastric secretions it is built up into solid compounds before digestion proceeds, which increase in density as the infant's secretions become stronger. Milk of all animals has this property and gradually strengthens the digestion of the young and fits it for its future work. It is because animals digest food in different ways and have different kinds of digestive tracts that milks are not alike. Alkalies added to cow's milk prevent the gastric secretion forming solid compounds with it and allow the milk to remain fluid in the stomach and readily to pass into the intestine. Gruel diluents allow the stomach to perform its function, but soften the solid curds so as to make them easily digested. Dextrinised or digested gruel diluents put the starch in a soluble condition and add to the proteid value of the food. The object of the present paper is to bring out and emphasise the biologic aspect of infant feeding which has heretofore been overlooked.

DISCUSSION.

Dr. Irving M. Snow: The members of the Academy have reason to feel well acquainted with Dr. Chapin. His book has been widely read and his methods are much employed by Buffalo physicians. He has given us this evening a very original presentation of his subject. There are many problems in the nutrition of the infant that are still unsolved. It seems that a baby cannot thrive without first receiving colostrum milk. Again, many babies are entirely unable to digest cow's milk. I do not understand why this is true. We have all noticed that children who have been fed on malted milk or condensed milk, if they survive to the age of three or four years, are often as well and strong as those who have been nursed by the mother. I am unable to account for this fact, also. On the other hand, it is quite true that the introduction of better methods of infant feeding has greatly reduced the death rate of infants in our cities. For this result, Dr. Chapin and other workers in this field of medicine deserve hearty thanks.

Dr. Dewitt H. Sherman: Dr. Chapin's plan is easily followed by those who have his ladle for measuring the milk. Unless the mother has some such instrument, I find that she is often in doubt as to the amount of milk to remove from the bottle. I have succeeded in simplifying this matter very much by asking the mother to remove the upper half when she wants to obtain an 8 per cent. of milk. The upper one-third of the bottle contains 12 per cent. milk, and, as a matter of convenience, I call the upper one-fourth of the bottle a 16 per cent. milk. In this manner, taking one-half, one-third or one quarter of the bottle and adding the proper diluents, the home modification of the milk is greatly simplified. Formulas are hard to remember, but if we remember the strength wanted in our combination, and the figures given above the problem is easier. It is not necessary to attempt to be more exact than is possible under the plan just outlined; we remember that the actual percentage formulas in vogue a few years ago did not give the satisfactory results that were theoretically called for.

Dr. W. G. Taylor: Some of my cases have apparently flourished on cow's milk, but have suffered severely from constipation. How would the doctor treat such cases?

Dr. G. H. A. Clowes: From the standpoint of the chemist, the laboratory is very unreliable for arriving at the proper mode of feeding children. I have never seen a better presentation of this question than that which Dr. Chapin has just given us. I wish to ask the doctor how we are to harmonise the statements in his table to the effect that increase of proteids in the diet of young animals leads to an increase in growth and strength, with the facts brought out in some of the recent experiments of Professor Chittenden in which he reduced the amount of proteid food and obtained a corresponding increase of strength in the subjects of his research. That portion of the proteid food which, instead of being utilised for the formation of tissue, is oxidised and goes to form heat energy is likely to produce an excess of amido bodies, and the like, which exert a detrimental action on nutrition. Can we not, therefore, use less proteid for children and get a stronger race? Another question relates to the proper amount of salt and other salines to be added to milk in order to bring it up to the normal percentage existing in the food of adults. How much salt should be given the infant, and how does it affect the production of hydrochloric acid in the stomach? The wide range in the ability of different children to assimilate the casein of cow's milk may be explained by the biologic reactions of different individuals toward the same proteid. We know that two carbohydrates,—as cellulose and starch,—can be easily differentiated by chemical tests. The various caseins, on the other hand, vary in their biologic rather than their chemical reactions.

When the proteids present in the serum of one animal are injected into an animal of another species, in the course of time the serum of this second animal is capable of precipitating the serum of the animal first employed and of all other animals of the same species, whilst animals of other species are only affected in a lesser degree. This differentiating reaction may frequently be effected when it is entirely impossible to show any chemical difference between the bodies in question.

Does Dr. Chapin think that this property of the proteids accounts for the variation in the ability of different children to assimilate certain proteids?

Dr. Chapin, in closing: Constipation is hard to correct, especially in bottle-fed infants. In my experience rolled oats have proven helpful in such cases. In reply to Dr. Clowes I would say, first, that Dr. Chittenden's experiments were on adults, while the question before us is how much proteid to give the baby. We must refer back to the composition of woman's milk and we find that it contains from 1 to 2 per cent. of proteid. It is a biologic fact that we cannot essentially alter the percentage of ingredients in the milk of a given animal, no matter what change we produce in its quantity. This is practically true of woman, although if a nursing woman is nervous the proteids may run somewhat higher and become harder to digest. This condition is improved by rest. The baby grows very rapidly. Growing bone and muscle require a considerable amount of proteid. During the entire period of nursing, the proteid content of the milk does not change, but the reaction of the baby's digestion does change. It is not well to feed a low percentage of proteid permanently; you are likely to produce a fat baby, deficient in bone and muscle. It is well for the doctor to bear in mind that he can not estimate the growth of a child by its weight alone. It is my practice to feel the baby as well as to weigh it. As to the use of vegetable proteids in infant feeding, we find that, unless they are perfectly broken up, they are not as easily digested as animal proteids, even when fed in the same percentage. In answer to the question concerning salt in the infant's food, I think that if the proteids are given in proper proportion the salts will not be deficient.

Owing to the lateness of the hour Dr. Bayliss desired to postpone the reading of his paper to a future meeting.

The academy adjourned at 10.25. Total attendance, 74.

---

### *Section on Medicine, May 9, 1905.*

REPORTED BY FRANKLIN W. BARROWS, M. D., Secretary.

THE regular meeting of the medical section was held Tuesday evening, May 9, 1905, at the academy rooms in the Public Library building, at 8.50 o'clock, the chairman, Dr. Allen A. Jones, presiding. The minutes of the last meeting were read and approved.

### PRESENTATION AND DISCUSSION OF CASES.

Dr. DeLancey Rochester: Some time last autumn a patient was sent from a neighboring town to the Buffalo General Hospital mainly for diagnosis of a disease of the knee joint. The case was diagnosticated as tuberculosis and after being treated for some time was sent home greatly improved, free from ulceration and discharge. The patient's father inquired at the time, and was instructed as to the nature of the affection and the proper care of the case. Recently a letter has been received from the father, a part of which I will read this evening. He complains that his milk trade has been ruined on account of this single case of tubercular joint disease in his home. This incident shows the unreasonable prejudice of many communities against this class of patients, and points the need of better information on the subject of the infectiousness of tuberculosis.

Dr. A. W. Bayliss: It would be well if the physician were more guarded in his statements concerning diagnosis. If, in this case, it had been called necrosis of the joint instead of tuberculosis the statement would have made less trouble.

Dr. ROCHESTER: As this case was sent to Buffalo largely for diagnosis, to relieve the doubts of the local doctors, it was necessary to call it by its real name.

ELECTION OF OFFICERS.

Dr. Lytle nominated Dr. Bayliss for the position of chairman. On motion of Dr. Rochester, seconded by Dr. Thoma, the secretary was instructed to cast the ballot of the section for Dr. Bayliss, who was elected.

Dr. Grosvenor nominated the present secretary to serve for another year. The secretary declined the nomination. Dr. Lytle nominated Dr. Taylor, who said he was unable to serve. Dr. Congdon nominated Dr. Colton, who also declined to serve. On motion of Dr. Rochester, seconded by Dr. Lytle, the chair was instructed to cast the vote of the section for Dr. Barrows, who was elected secretary.

Dr. ALBERT T. LYTLE read a paper entitled,

THE NURSE OR THE DOCTOR?

(*Author's Abstract.*)

The author gave a historical review of the development of the present system of education of nurses, stating that, while hospitals were known for a considerable period before the Christian era, yet the first European public hospital was erected in Rome in 380. He said that nursing, prior to 1835, was carried on by religious orders like that of the Sisters of Charity of today; that the modern idea of trained nurses was evolved from the inception of a hospital at Kaiserswerth, Germany, by Pastor Fliedner; that instruction for nurses was systematised by Florence Nightingale, who, in 1853, established a training school in Saint Thomas's Hospital, London, which became the nucleus of the present elaborate system of training; that in America special training dates back to 1863, but was not fully developed until 1873; that 1879 saw the establishment of the first training school in Buffalo; that since 1873 nearly every institution caring for the sick had established some sort of a nurse training school until more than 552 such institutions now exist in the United States.

He called attention to the extensiveness of the movement as evidenced by the formation of local, state, national and international associations, congresses and the publication of weekly, monthly and quarterly periodicals exclusively for the benefit of this profession. He compared the courses of training offered by the various schools throughout the world and practically divided them into two groups, one of which gives theoretical prior to practical training, while the other gives theoretical instruction interspersed throughout the entire course of practical work; that in general the time spent is three years. He said that the books recommended for use, the character of the class work and the lectures, and the practical work in well equipped hospitals, indicated that nurses received an education in medicine but little inferior to that given in many medical colleges.

He called attention to the remarkable demand for trained nurses, which had practically increased ten fold in the past ten years; to the probability that the average annual income of the trained nurse was in the neighborhood of $450 exclusive of living, while $1,500 would be an extreme remuneration; to the fact, that untrained women were rushing into the profession owing to the pernicious advice of well meaning friends,—especially pastor and physician. Dr. Lytle then summed up the state registration laws, hinted at the advantages to be derived therefrom by nurses, and stated that eight states had adopted laws governing the registration of nurses. He spoke of the excellent quality of the theoretical work given by a few of the correspondence schools; described the four year course given by a Massachusetts school wherein nurses pay for their education like any other students seeking technical attainment and quoted opinions severely criticising present school systems and the payment of nurses for their services in the hospitals while in training.

In conclusion, he said that a comparison of the opportunity for medical experience, which a course in nursing offers to the student as compared to that of a course in medicine, considering the textbooks recommended for nurses, would lead many physicians to think that a nurse received a better and more practical training for the practice of medicine at a much less expense than does the student who aspires to the degree of M. D.; that owing to the character of the education as indicated by the curriculums reviewed, it was simply a matter of justice to the graduate nurse to assume that she should consider from the fulness of her education that she had the right to use the acquired knowledge in benevolent and romantic aid to the sick,—i. e., to practise medicine; that because of the intimate relation of nurse to patient the opportunity to diagnosticate and to treat diseased conditions which should naturally fall to the care of the doctor was of very frequent occurrence and that the nurse was taking advantage thereof; that the rapid and comprehensive increase in educational requirements and in the technical equipment of nurses would naturally and justly cause them to assume the position of competitors and that this would be but the boomerang blow which hospitals and physicians had incurred because on the plea of philanthropy and for the benefit of the innocent public, they had used the trained nurse and the training of the nurse for purely mercenary ends and personal advancement.

The author prophesied that the effect of this competition would be good in that it would drive out of the field the fakir, the quack, the osteopath, the Christian scientist, the clairvoyant, the patent medicine man, the advertiser and a host of like frauds, and that it would compel the physician to be more careful and to keep abreast of the advances in his profession.

### DISCUSSION.

Miss Sylveen Nye: I wish to embrace this opportunity to emphasise as a fact the remarks of Dr. Lytle to the effect that the medical and nursing professions are dependent and interdependent. Much more than appears to the laity, is the nursing dependent upon the medical profession. Nurses must practise in harmony and conjunction with physicians if their mission is to be reached with success.

In 1900 a paper, prepared by myself, was published in the *Trained Nurse and Hospital Review* in which I advocated the proposal of a bill to the New York state legislature that should incorporate the following principles:

1. Uniform entrance examinations.
2. Uniform curriculum.
3. Uniform final examinations conducted by the regents.
4. That no hospital be allowed to maintain a training school for nurses unless it could in its own school, or through combination with another, furnish a general training.

I realised that this would necessitate radical changes in many hospitals and could not be accomplished in a short time. It has never been my idea to institute any change in general hospital management that would result otherwise than in ultimate benefit to the hospital as well as to the nurse.

In the winter of 1902 and 1903 at my instigation, a bill endorsed by the Buffalo Nurses's Association was introduced in the New York state legislature by Assemblyman Nye, of Schuyler, which provided that every nurse who had graduated from a hospital existing in conformity with the laws of its state, might register. Our contention was that the state had not the right to permit hospitals to establish training schools, graduate pupils, issue diplomas and then by statute bar them from the rights of a trained nurse. The passage of such a bill would have met with immediate recognition from all nurses and would have withheld the power of a created board of examiners passing upon the qualifications of a nurse already graduated, and legalised to practise by our existing laws. The first registration should have been made simple and easy; then should have followed, in due time, another bill requiring uniform entrance examinations, uniform curriculum, and uniform final examinations. In a few years the matter of hospitals furnishing a general training, by combining, would have been of comparatively easy solution.

I believe that physicians should be included in the board of examiners. Under existing conditions it is difficult for one to know just what are the present standards of nursing. To me it seems that the present New York State law regarding the registration of nurses is not a success. While it has, doubtless, been an incentive and has improved conditions in many training schools, many other hospitals have resorted to subterfuges and arrangements which remind one of the results following the passage of the Raines liquor bill, when every saloon became a hotel.

I thank Dr. Lytle for his reference to the overworked nurse. In the training school from which I graduated we were constantly and rightfully instilled with our duty to the physician and to the hospital. Was it strange that the intelligent woman occasionally questioned if there was an equal sense of responsibility of the duty of the hospital and the doctor to the pupil nurse?

I would like also to use this opportunity to protest against the average address to graduate nurses. If any of the doctors present have such duties to perform I beg that they will not make it an occasion to remind the laity of the many faults of which the nurse might become guilty. Usually these things are but a reiteration of what she has been taught from the moment of her entrance to the hospital. Such remarks usually suggest to the people present so many wrong things that a nurse might do, and when the occasion comes for a nurse to be employed in the family it is assumed that she will fall necessarily into the errors against which the doctor has warned her at the time of her graduation.

I look with favor on the movement in Boston for bringing the physicians and nurses closer together. Too many nurses feel that after leaving the training school their education is finished. I believe that nurses' organisations should advocate and encourage a plan of study along the lines of a University Extension Course, assisting the members materially in self improvement and advancement.

In behalf of the Buffalo Nurses' Association I thank Dr. Lytle for his paper and the Buffalo Academy of Medicine for its courtesy in permitting us to be present on this occasion.

Dr. LYTLE, in closing: I have greatly enjoyed the work of preparing this paper. I am surprised at the value of the mental training in preparation for nursing received by women.

[NOTE BY THE EDITOR.—In relation to this subject, the address by Dr. F. Park Lewis, published elsewhere, page 781, will be of interest.]

Dr. A. W. BAYLISS read a paper on

THE TREATMENT OF DISEASE BY MEANS OTHER THAN DRUGS.

*(Abstract.)*

This paper deals with what may properly be called physiological therapy, or means employed to assist the return from a pathological to a physiological condition. The body of the athlete illustrates the effects of exercise and massage, as they might be applied to the treatment of certain muscular conditions. Chronic constipation which has resisted all the cathartic drugs often yields to electricity and vibration aided by mild laxatives. In shock, during or following operation, the most efficient relief comes not from drugs but from physical and mechanical means, such as stimulation of the skin by heat, of the heart and respiratory centers by electricity, the introduction of normal salt solution and in extreme cases massage of the heart.

I am in favor of using drugs only when we cannot succeed without them. When we want diaphoresis why not produce it by direct action on the skin instead of giving a drug that acts on the central nervous system and depresses the patient?

Physiological therapy sometimes misses its aim because of faulty apparatus or faulty application. Many of the appliances now used for generating electricity are worse than useless. The proprietor of a sanitarium recently exhibited to me, with sincere pride, his ultra violet apparatus, which consisted of about six feet of flexible cord with a common incandescent bulb colored purple. He expected to benefit diseased tissues by holding this lamp in front of them. A Finsen light must carry at least 40 amperes. Those who use such agencies must first understand the principles on which they work and their action on normal as well as on diseased tissues.

It is more than unjust to your patient to begin a course of electric treatment without carefully outlining it in your own mind.

For electrical treatment one must have an outfit for supplying the constant and induced currents. For the galvanic current one needs at least 30 cells, a rheostat and a milliampere meter. The coils for the induced current should be interchangeable and made of different sizes of wire capable of being tapped at different points for producing at will sedative or stimulant effects.

Massage was practised in the times of Plato and Hippocrates. The term includes many forms of exercise applied to the patient either by means of the hands or by some mechanical contrivance. Our modern machines accomplish more in a given time than can be done with the hand and admit of all the variations necessary in the treatment of delicate superficial structures or deeper tissues. Vibration is not adapted to conditions that are not localised; it will not take the place of the gymnasium properly utilised under a competent director.

I believe that one reason why the original type of Finsen light is not a success in America is that the American physician has not the time to devote to this form of treatment. Dr. Finsen asserts that 94 per cent. of cases of lupus and rodent ulcer are cured by his treatment. But his instrument carries a current of 40 to 80 amperes and a treatment lasts one hour. Why should we expect the same results from 10 or 15 minutes' treatment with a current of one to three amperes?

Many physicians are inclined to discard *x*-ray treatment after seeing one failure. If the surgeon, after his first fatal case of appendectomy or tumor operation, were to conclude that surgical treatment of all such cases is a failure, he would be on the same level with many experimenters in *x*-ray treatment. Most of the cases coming to the *x*-ray specialist are too far advanced to get relief. They should begin treatment before ulceration has set in. I have yet to see the case of malignancy that came to me in its first stage and was not benefited or cured by the *x*-rays.

Much could be said on the different kinds of baths and their uses. There are many cases in which some form of bath would be used to advantage, were not this branch of therapy so generally neglected. Why should not our colleges add to the medical curriculum the branch of physiological therapy?

### DISCUSSION.

Dr. Rochester: I thank the doctor for presenting to the academy a branch of treatment that is greatly neglected. For ten years I have been in the habit of writing out directions for my patients instead of prescriptions. It often surprises and amuses the patient to see so much stress laid on matters of habit and so little dependence placed on drugs. I advise my students never to prescribe drugs when they can employ other means to the same ends. If we do not know how to use the apparatus described in the doctor's paper we should not try to use it, but should send the patient to the specialist.

Dr. Congdon: What means would Dr. Bayliss use for stimulating a patient after operation?

Dr. Bayliss, in closing: In extreme cases where, following operation, the respiration and heart are failing, medicine is of very little use. In such cases I would use mechanical means of stimulation.

The meeting adjourned at 10.15 o'clock. Attendance, 53.

